# Fano-Resonance in Hybrid Metal-Graphene Metamaterial and Its Application as Mid-Infrared Plasmonic Sensor

**DOI:** 10.3390/mi11030268

**Published:** 2020-03-04

**Authors:** Jianfa Zhang, Qilin Hong, Jinglan Zou, Yuwen He, Xiaodong Yuan, Zhihong Zhu, Shiqiao Qin

**Affiliations:** College of Advanced Interdisciplinary Studies, National University of Defense Technology, Changsha 410073, China; qlhong95@126.com (Q.H.); 18380597763@163.com (Y.H.); x.d.yuan@163.com (X.Y.); zzhwcx@163.com (Z.Z.); sqqin8@nudt.edu.cn (S.Q.)

**Keywords:** fano resonance, graphene, plasmonic sensor

## Abstract

Fano resonances in nanostructures have attracted widespread research interests in the past few years for their potential applications in sensing, switching and nonlinear optics. In this paper, a mid-infrared Fano resonance in a hybrid metal-graphene metamaterial is studied. The hybrid metamaterial consists of a metallic grid enclosing with graphene nanodisks. The Fano resonance arises from the coupling of graphene and metallic plasmonic resonances and it is sharper than plasmonic resonances in pure graphene nanostructures. The resonance strength can be enhanced by increasing the number of graphene layers. The proposed metamaterial can be employed as a high-performance mid-infrared plasmonic sensor with an unprecedented sensitivity of about 7.93 μm/RIU and figure of merit (FOM) of about 158.7.

## 1. Introduction

In the past decade, the so called Fano resonance—a type of resonance originated from the constructive and destructive interference of a narrow discrete resonance with a broad spectral line or continuum—has attracted wide spread research interests in the nanophotonics community [1]. Fano resonances have been observed in various dielectric or plasmonic nanostructures such as metamaterials [2,3,4,5,6], oligomers [7], nanocavities [8,9,10] and so on [11,12,13]. The sharp variation of the scattering profile by Fano resonances leads to a variety of applications in optics such as sensing, switching and nonlinear devices. Particularly, Fano resonances in plasmonic nanostructures are sensitive to the changes of local environment and a small perturbation can induce dramatic change of scattering profiles [14]. Thus, Fano-resonant plasmonic structures, in combination with the appropriate chemical and bio-markers, could enable the development of label-free chemical and bio-sensors [15,16,17,18,19,20].

Traditionally, plasmonic nanostructures are built on noble metals such as silver and gold and they work mainly in the visible and near-infrared (IR) ranges. Meanwhile, graphene has recently been rising as a building block for plasmonic devices in the mid- and far-IR ranges [21,22,23]. Graphene plasmons show relatively low losses, high spatial confinement and incomparable tunability by chemical or electrostatic doping [24,25], providing an versatile platform for tunable infrared devices [26,27], mid-IR sensing [28,29,30,31], photodetectors [32,33] and other applications. Hybrid metal-graphene structures have also been studied [34]. The plasmonic resonances of metallic nanostructures can be employed to enhance light-graphene interactions in the visible and IR ranges [35,36] while graphene provides an ideal material to tune the optical properties of metamaterials [34,37,38,39]. Due to the different plasmonic properties of metal and graphene, a hybrid metal-graphene structure could be designed to show multi resonances in ultra-broadband spectral ranges from near to mid-IR ranges [40]. Their resonances can also coupling with each other, exhibiting interesting resonant behavior such as Fano resonances [34,41,42].

In this paper, a hybrid metal-graphene metamaterial with a Fano-like resonance in the mid-IR range is proposed. The Fano resonance arises from the coupling of the narrowband plasmonic resonance of the graphene nanostructure to the broadband resonance of the metallic structure. Its linewidth is narrower than that of the graphene plasmonic resonance and the resonance strength can be controlled by changing the number of graphene layers. Its potential as a mid-IR refractive index sensor is studied.

## 2. Results and Discussion

**Figure 1:**
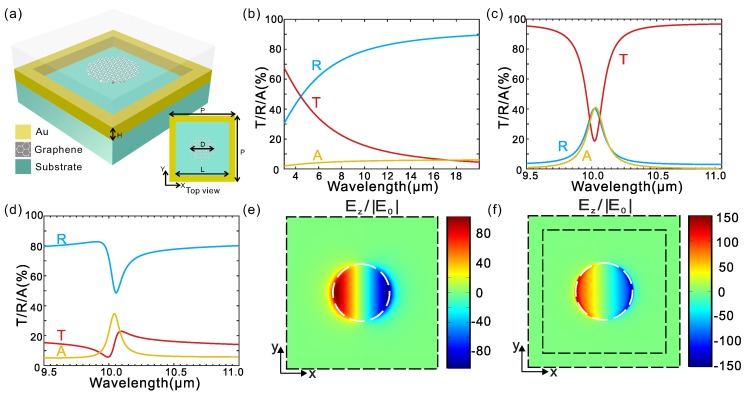
A hybrid metal-graphene Fano-resonant metamaterial. (**a**) Schematic of the hybrid metamaterial. (**b**–**d**) Simulated spectra of transmission, reflection and absorption for different combination of nanostructured gold film and graphene nanodisks including nanostructured gold film without graphene nanodisks (**b**), graphene nanodisks without the nanostructured gold film (**c**) and nanostructured gold film enclosing with graphene nanodisks (hybrid metamaterial) (**d**). (**e**,**f**) Distributions of local electric fields in the z-direction at the resonance wavelength for graphene nanodisks at ∼10μm and the proposed hybrid metamaterial at ∼10.05μm, respectively. The fields are normalized to the field amplitude of the incident wave ($E_{0}$) and plotted at the x-y plane that is 5nm above the graphene nanodisks. The x-polarized light impinges on the top side of the structure at normal incidence.

Figure 1a shows the schematic of the hybrid metamaterial. The structure comprises a cover layer, a metallic grid (gold film with periodic holes) enclosing with graphene nanodisks right in its middle and a semi-infinite substrate. The graphene nanodisks are assumed to locate right in the middle of the holes (at the same height of the top surface of the metallic grid). The period of a unit cell is P=800 nm and length of the square hollow in the gold film is d=300 nm. The height of the gold layer is H=30 nm and the diameter of graphene nanodisks is d=300 nm. The structure is excited by a x-polarized wave at normal incidence.

For the metallic structure without graphene, the calculated spectra are shown in Figure 1b. The plasmonic resonance is broadband and the resonance peak in the short wavelength range is not shown here. For the periodical array of graphene nanodisks alone, the optical spectra are shown in Figure 1c. There is a plasmonic dipolar resonance (see Figure 1e) at the wavelength of around 10 μm and the full width at half maximum (FWHM) of the resonance is about 0.16 μm. For the hybrid metal-graphene metamaterial, the coupling between the broadband plasmonic resonance of the metallic nanostructure and the narrowband plasmonic dipolar resonance of the graphene nanodisks leads to a sharp Fano resonance at around 10.04 μm with a FWHM of about 0.05 μm (Figure 1d,f).

**Figure 2:**
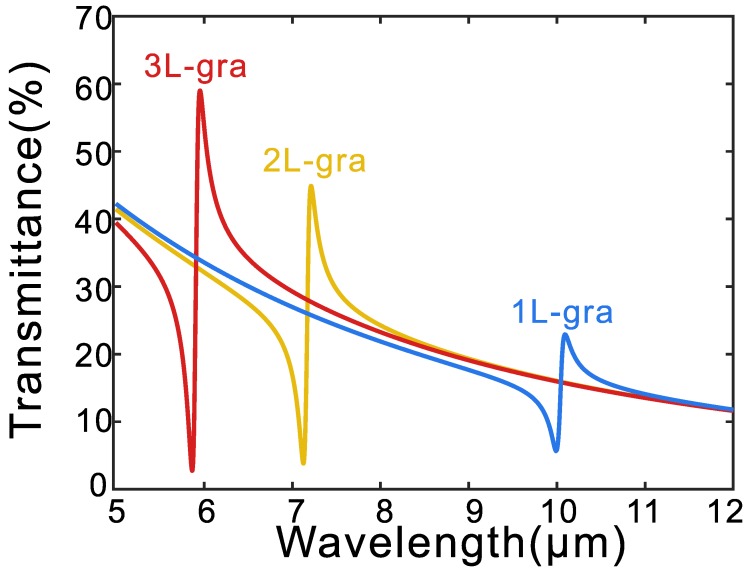
Simulated spectra of transmission for (**a**) Graphene nanodisks and (**b**) The hybrid metal-graphene metamaterial with different layers of graphene.

An effective way to manipulate the mid-IR resonance is controlling the Fermi energy of graphene. However, it is technically challenging to realize Fermi engergy higher than 1 eV. Another way to enhance the plasmonic responses of graphene is using stacked graphene instead of monolayer. Previous studies have shown that infrared plasmonic response of a graphene multi-layer stack is analogous to that of a highly doped single layer of graphene [43]. For simplification, we assume that multilayers of graphene are stacked without separation and it can be replaced by an equivalent layer having the sum of the conductivity of each layer. Figure 2 shows the simulated transmission spectra of the hybrid metal-graphene metamaterial with different layers of graphene. With the increase of graphene layers, the resonance blue shifts and the intensity increases. As the graphene layer increases from 1 to 2, the Fano resonance blue shifts from 10.04μm to 7.17μm and amplitude of resonance in transmission increase from 17.3% to 41.1%. Furthermore, the Fano resonance blue shifts to 5.91μm and amplitude of resonance in transmission increases to 56.3% for 3 layers of graphene.

**Figure 3:**
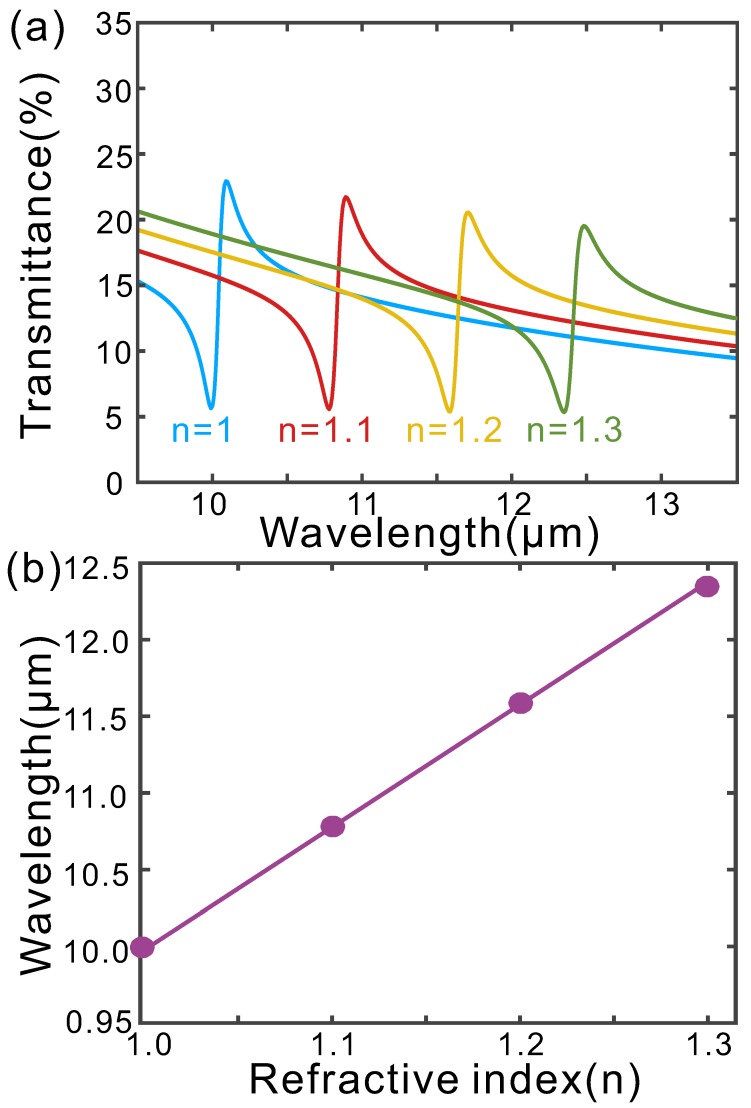
(**a**) Calculated transmittance of the hybrid metal-graphene metamaterial with a cover layer of different refractive indices. (**b**) Wavelengths of the transmittance dips as a function of the cover layer’s refractive index.

The sharp Fano resonance of our proposed hybrid graphene-metal metamaterial can increase the figure of merit (FOM, the sensitivity value divided by FWHM) of sensors and is suitable for mid-IR plamsonic sensing. To evaluate its sensing ability, we calculate the transmission spectra of the hybrid metamaterial when it is covered by a semi-infinite layer with different values of refractive indices (Figure 3). As the refractive index of the cover layer (including the medium in the holes of the gold film) increases from 1 to 1.3, the resonance wavelength red shifts from 10.04μm to 12.42μm, corresponding to a linear sensitivity of about 7.93μm/RIU (Figure 3b) and FOM of about 158.7.

**Figure 4:**
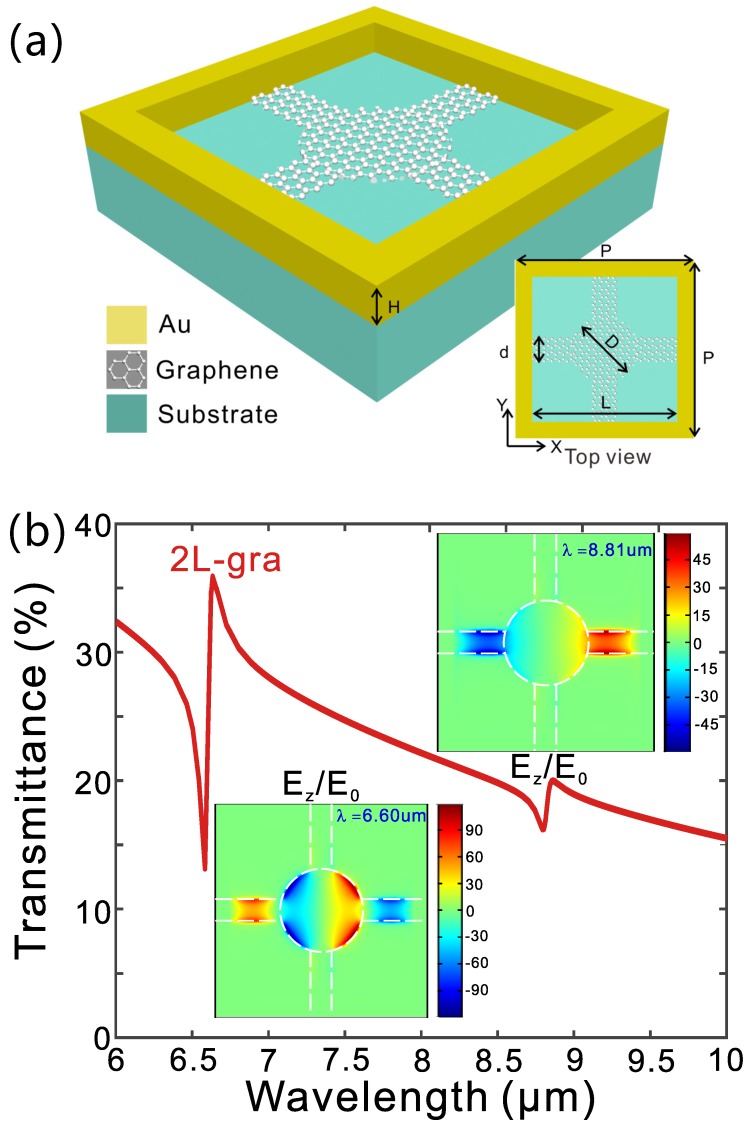
(**a**) Schematic of a modified hybrid metal-graphene metamaterial. (**b**) Simulated spectra of transmission. The insets are field distributions at the two resonances which are normalized to the field amplitude of the incident wave ($E_{0}$) and plotted at the x-y plane that is 5nm below the graphene nanodisks.

In the above, we have shown that the hybrid graphene-metal metamaterial exhibits sharp Fano resonance in the mid-IR range and it can be employed for high-performance sensing. The discussed structure possess several drawbacks in practical realization. First, the graphene nanodisks are electrically isolated and this makes it difficult for electrostatic doping and dynamic modulation of Fermic energy (electrostatic doping with ion-gel is possible but the cover layer will affect its sensing applications). So we may need to employ chemically doped graphene. Secondly, we have assumed that the graphene film is located at the same height of the top surface of the metallic grid which is difficult to fabricate. In order to solve these problems, modified structures can be employed. As an example, we show a hybrid metal-graphene metamaterial where graphene is located directly on the surface of the substrate and the graphene is electrically connected (Figure 4a). Such a structure can be fabricated with standard nanofabrication technique where CVD grown graphene can be transferred to the substrate and patterned by electron beam lithography (EBL). Then the metallic nanostructure can be fabricated on graphene by aligned EBL along with a lift-off process. The simulated spectra of transmission for the hybrid metamaterial with two layers of graphene are shown in Figure 4b. Similar to the spectra in Figure 2, there is a sharp Fano resonance at around 6.60μm. Besides, an additional resonance appears at around 8.81μm. These two resonances arise from the coupling between the broadband plasmonic resonance of the metallic nanostructure and the narrowband plasmonic resonance of the graphene nanodisks and that of the graphene cross arms (see insets of field distributions in Figure 4).

## 3. Materials and Methods

The numerical simulations are conducted using a fully three-dimensional finite element technique (COMSOL Multiphysics, Stockholm, Sweden). In simulations, the monolayer graphene sheet is modeled as a conductive surface [44,45] and the transition boundary is used for it. Optical conductivity of graphene can be derived within the random-phase approximation (RPA) in the local limit as below [46,47].

(1) $$\begin{array}{cc} \sigma (\omega )= & \frac{2e^{2}k_{B}T}{\pi \hbar^{2}}\frac{i}{\omega +i\tau^{-1}}\ln\left[2\cosh\frac{E_{f}}{2k_{B}T}\right]+\\ \; & \frac{e^{2}}{4\hbar }[\frac{1}{2}+\frac{1}{\pi }\arctan\left(\frac{\hbar \omega -2E_{f}}{2k_{B}T}\right)-\\ \; & \frac{i}{2\pi }\ln\frac{{\left(\hbar \omega +2E_{f}\right)}^{2}}{{\left(\hbar \omega -2E_{f}\right)}^{2}+{\left(2k_{B}T\right)}^{2}}], \end{array}$$

where $k_{B}$ is the Boltzmann constant and T=300K is the temperature; ω is the frequency of incident wave; τ denotes the carrier relaxation lifetime; $E_{f}=0.9\;\mathrm{eV}$ is the Fermi energy. And We have $\tau =\mu E_{f}/eV_{F}^{2}$. $V_{F}=10^{6}\;m/s$ is Fermi velocity. The mobility is $\mu =10,000\;{\mathrm{cm}}^{2}/\left(V\cdot s\right)$, which could be realized by chemical or electrostatic doping [48,49].

The substrate is assumed to be lossless with the refractive index n=1.4 and the cover layer is also semi-infinite with refractive index n=1 at the beginning. The permittivity of Au is described by the Drude-Lorentz dispersion model with plasma frequency $\omega_{p}=1.37\times 10^{16}\;s^{-1}$ and the damping constant $\omega_{\tau }=4.05\times 10^{13}\;s^{-1}$.

## 4. Conclusions

In summary, a mid-infrared Fano-like resonance in a hybrid metal-graphene metamaterial has been studied. The Fano resonance arises from the coupling of the broadband resonance of the metallic nanostructure and the narrowband plasmonic resonance of the graphene nanostructure. It is sharper than plasmonic resonances in pure graphene nanostructures. The resonant strength can be effectively enhanced by increasing the layer numbers of graphene. The sensing properties of the proposed metamaterial are studied and it shows a sensitivity of about 7.93μm/RIU. Such a sensitivity is higher than most of reported graphene plasmonic sensors in the mid-IR [28,50] while the reduced line width of the Fano resonance leads to an further increased FOM of about 158.7. The proposed concept can be employed for various modified structures. As an example, we have shown a modified hybrid graphene-metal metamaterial which is relatively easier to fabricate. The simulated transmission spectra show similar Fano resonant responses. This work may stimulate the study on Fano resonances in hybrid plasmonic structures and find applications in mid-IR sensing and others areas.
